# Association between Mediterranean‐dietary approaches to stop hypertension intervention for neurodegenerative delay diet and biomarkers of oxidative stress, metabolic factors, disease severity, and odds of disease in rheumatoid arthritis patients

**DOI:** 10.1002/fsn3.4055

**Published:** 2024-03-12

**Authors:** Mahdieh Safaei, Sorayya Kheirouri, Mohammad Alizadeh, Amir‐Hossein Pirovi

**Affiliations:** ^1^ Department of Nutrition Tabriz University of Medical Sciences Tabriz Iran; ^2^ Nutrition Research Center Tabriz University of Medical Sciences Tabriz Iran; ^3^ Department of Rheumatology, School of Medicine Tabriz University of Medical Sciences Tabriz Iran

**Keywords:** lipid profile, metabolic factors, MIND diet, oxidative stress, rheumatoid arthritis

## Abstract

This research aimed to examine the association between the following Mediterranean‐DASH Intervention for Neurodegenerative Delay (MIND) dietary pattern and oxidative stress indicators, metabolic factors, disease activity, and the odds of disease in patients with rheumatoid arthritis (RA). In this cross‐sectional study, we included 101 patients with RA and 101 healthy individuals. The MIND diet score was measured using a semi‐quantitative Food Frequency Questionnaire (FFQ) with 147 food items. Total capacity antioxidant (TCA), superoxide dismutase (SOD), glutathione peroxidase (GPX), and malondialdehyde (MDA) serum concentrations were evaluated by ELISA, and the disease severity was measured regarding the disease activity score 28 (DAS‐28) criteria. The average score of the MIND diet was substantially lower in the RA subjects than in the healthy people (*p* < .001). Individuals with a higher MIND diet score had lower odds of RA than those with a low score (*p* < .001). There was no remarkable link between the MIND diet and oxidative stress factors (*p* > .05). A reverse association was found between the MIND diet score and disease activity (*p* < .05). The MIND diet was significantly and negatively correlated with triglycerides, low‐density lipoprotein cholesterol, total cholesterol, fasting blood glucose, and hemoglobin A1C. There was a positive association between the diet and high‐density lipoprotein cholesterol. The findings indicate that following the MIND diet may decrease disease activity and the odds of RA. Also, high adherence to the MIND diet may improve the lipid profile and blood glucose status in RA patients.

## Graphical abstract

The findings indicate that following the MIND diet may decrease disease activity and the odds of rheumatoid arthritis. Also, it may improve the status of the lipid profile and blood sugar in these patients.

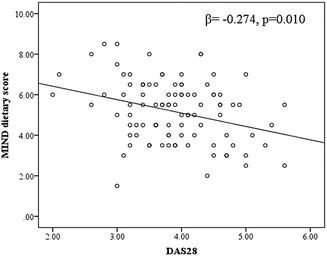

## 1 INTRODUCTION

Rheumatoid arthritis (RA) is a long‐term autoimmune disorder that primarily manifests as inflammation in the joints. This disease often affects the hands and wrists. The main symptoms of this disease include joint pain, morning stiffness, and joint swelling (Smolen et al., 2018). In patients with RA, synovial hyperplasia and destruction and sensitivity of the joints may harm physical, emotional, and social functioning. RA causes systemic disorders such as cardiovascular, pulmonary, and other skeletal disorders and increases mortality (Kaplan, 2010). About 1% of the global population has RA, which is more prevalent in the fourth and fifth decades of life and influences women more than males (Moshayedi et al., 2022).

The exact cause of RA is not known. However, genetic susceptibility and environmental triggers such as smoking, unhealthy lifestyle choices, nutritional risk factors, air pollution, dust, and infections play a role in causing the disorder (van der Woude & van der Helm‐van Mil, 2018). The information available suggests that oxidative stress contributes to the pathophysiology of RA (Wang et al., 2022). The enhancement in the activity of the immune system is associated with an increase in the production of free radicals, which can damage the joint tissue and lead to extra‐articular problems such as cardiovascular complications (Zamudio‐Cuevas et al., 2022). The production of free radicals has a destructive effect on surrounding tissues and affects the quality of life of people with RA (Phull et al., 2018). According to the findings of a recent investigation, food and nutrition play a vital role in controlling oxidative stress (Tan et al., 2018).

Evidence shows that diet and nutrients, as environmental factors, may be involved in RA development (Alpízar‐Rodríguez et al., 2020). Since people do not consume nutrients individually and intake nutrients in the form of a diet, most recent investigations have focused on dietary patterns, which examine the general effects of diet, as a novel method to investigate the correlation between diet and the incidence of chronic diseases, including RA (Forsyth et al., 2018; Ghaseminasabparizi et al., 2021).

The Mediterranean‐DASH (Dietary Approaches to Stop Hypertension) Intervention for Neurodegenerative Delay (MIND) diet was first recognized as a dietary pattern that protects the brain and improves cognitive diseases. The MIND diet is a combination of the dietary patterns of the Mediterranean and DASH. The diet emphasizes the consumption of plant foods and limits the consumption of animal‐based meals and foods high in adverse fatty acids. Specifically, the MIND dietary pattern is characterized by the consumption of berries and plants (Morris, Tangney, Wang, Sacks, Barnes, et al., 2015). Previous investigations have reported that following the MIND diet reduces the incidence of dementia (Koch & Jensen, 2016). Furthermore, several recent investigations found positive effects of the MIND diet on the improvement of cardiovascular diseases (Song et al., 2023), obesity (Aminianfar et al., 2020), and metabolic syndrome (Mohammadpour et al., 2020). There is no research on the link between RA and the MIND diet.

Considering the impact of nutrition in the prevention or incidence of RA and the limited available studies regarding the association between MIND dietary patterns and RA diseases, the present research, for the first time, was directed to research the correlation between the MIND diet with oxidative stress indicators, metabolic factors, disease severity, and the odds of disease in RA patients.

## 2 MATERIALS AND METHODS

### 2.1 Participants

The present cross‐sectional investigation was accepted by the ethics board of Tabriz University of Medical Sciences (ethical number: IR.TBZMED.REC.1400.1149). Participants with newly diagnosed RA disease were selected by convenience sampling from those who attended the rheumatology clinic of Imam Reza Hospital in Tabriz City, East Azerbaijan, Iran. The clinical diagnosis of RA was done by a rheumatologist using the American European College of Rheumatology (ACR) criteria. ACR/EULAR scores included the number and location of involved joints (score range 0–5), serological anomaly (score range 0–3), enhanced acute‐phase action (score range 0–1), and symptom time (score range 0–1). Obtaining a minimum score of 6 and synovial hyperplasia in at least one joint unrelated to another disease is the criterion for a definitive diagnosis of RA. Some healthy people (*n* = 101) were also chosen from those referred to the nutrition clinic for routine health examinations. Individuals with renal failure, heart disease, hyperthyroidism, cancer, any bone and joint disease, and energy intake of less than 800 or higher than 4200 kcal/day, regardless of whether they were in the RA group or healthy group, were excluded from the research. Each of the subjects expressed their consent to participate.

### 2.2 Anthropometric measurements

The participant's weight was evaluated to the nearest 0.1 kg using an electronic scale while wearing a light dress and without shoes. The height was measured to the nearest 0.5 cm. The formula for computing body mass index (BMI) is body weight (kg)/height^2^ (m). A non‐stretch tape was used to measure waist circumference from the distance between the anterior upper pelvic spine and the last rib after a conventional exhalation, with no pressure on the body surface.

### 2.3 Determination of RA severity

The severity of the disease was calculated with the valid Disease Activity Score of 28 Joints (DAS‐28) index, which included the tender and swollen joint counts (TJC and SJC), general health (GH), which is measured using a 100 mm visual analog scale (VAS), and the C‐reactive protein (CRP) (mg/L) concentration as an acute phase reactant, based on the below formula:

$$\mathrm{DAS}-28=0.56^{*}\surd \mathrm{TJC}28+0.28^{*}\mathrm{SJC}28+0.70^{*}\mathrm{Ln}\;\left(\mathrm{CRP}\right)+0.014^{*}\mathrm{VAS}$$

### 2.4 Dietary assessment

The usual food intake of the persons in the past year was investigated utilizing a semi‐quantitative food frequency questionnaire (FFQ) with 147 food items. The Persian version of the FFQ has already been evaluated concerning reliability and validity (Mirmiran et al., 2010). The reported values of foods were converted to grams using the household scale guide. Finally, the amount of energy and macronutrients received for each food item was analyzed using the IV Nutritionist software (Nutritional Database Manager 4.0.1, USA).

### 2.5 MIND diet score

The scoring of the MIND diet was done utilizing the method of Morris, Tangney, Wang, Sacks, Bennett, and Aggarwal (2015). In this method, 15 food items were assessed. People were ranked into three categories based on their intake of good foods for the brain (such as olive oil, fish, whole grains, berries, leafy greens, other vegetables, nuts, legumes, and poultry). The third part received 1 point, the second part received 0.5 points, and the first part received 0 points. The classification was also done for the intake of cheese, butter, margarine, red meat and its products, instant fried meals, and sweets, and the scoring was considered inversely for them. The amount of wine consumed was not considered in the score computation because of the absence of reports in our dataset. Therefore, each participant had a score between 0 and 14. All groups were adjusted for energy before calculating the MIND score.

### 2.6 Lipid profile and fasting blood glucose

Triglyceride (TG), total cholesterol (TC) levels, and high‐density lipoprotein cholesterol were measured with autoanalyzer biochemical tools and enzymatic kits (Pars Azmoun, Tehran, Iran). Then, low‐density lipoprotein cholesterol was computed using the Friedwald formula (Scharnagl et al., 2001). Fasting blood glucose (FBG) level was measured by the glucose oxidase method using a commercial kit (Pars Azmoun, Tehran, Iran). Hemoglobin A1c was measured by the HPLC method. Serum insulin levels were measured using an ELISA kit (Navand Salamat, Iran).

### 2.7 Stress oxidative markers

Five millilitre of fasting (10–12 h) blood was collected from the participants. Serum samples were stored at −80°C until biochemical analysis. Serum glutathione peroxidase (GPx) activities, total antioxidant capacity (TAC), and superoxide dismutase (SOD) levels were measured by ELISA kits (Navand Salamat, Iran). Serum malondialdehyde (MDA) level was assessed using a peroxidation assay kit (Nalondi™, Navand, Salamat Co., Urmia, Iran).

### 2.8 Data analysis

The data were analyzed using SPSS Model 22 software (SPSS Inc). The Kolmogorov–Smirnov test was used to check the normality of the data. Quantitative data were indicated as mean ± standard deviation and qualitative data as frequency (%). To compare the two groups, Mann–Whitney or independent sample tests were used. Odds ratios (95% confidence intervals) were obtained utilizing binary logistic regression in the crude and multivariable‐adjusted models. Linear regression was applied to check the correlation between different factors. *p* values less than .05 were considered substantial for all tests. Age, gender, BMI, smoking, education, physical activity, and medication use were considered confounders.

## 3 FINDINGS

### 3.1 Participant's characteristics

The demographic and anthropometric characteristics and the score of the MIND diet of the participants are presented in Table 1. The average age of the participants was remarkably higher in the RA cases than in the healthy group (*p* < .001). The percentage of people with low education levels was substantially higher in the RA cases than in the healthy group (*p* < .001). Body weight (*p* = .003), BMI (*p* = .005), and waist circumference (*p* = .049) were considerably higher in the RA group compared with the healthy group. The mean score of the MIND diet was noticeably lower in the RA group than in the healthy group (*p* < .001). Other demographic variables (including gender, marriage status, cigarette use, and alcohol intake) did not show meaningful differences between the two groups.

**TABLE 1:** Demographic characteristics of participants.

Variable	Participants		*p*‐value
	RA patients (*n* = 101)	Healthy people (*n* = 101)	
Age^a^	44.79 ± 9.05	40.93 ± 7.85	<.001
Gender^b^			
Men	28 (27.7%)	19 (18.8%)	.091
Women	73 (72.3%)	82 (81.2%)	
Marriage status^b^			
Single	14 (13.9%)	12 (11.9%)	.417
Married	87 (86.1%)	89 (88.1%)	
Education level^b^			
Illiterate	22 (21.8%)	4 (15.4%)	<.001
Under diploma	44 (43.6%)	19 (30.2%)	
Diploma	15 (14.9%)	25 (24.8%)	
Educated	20 (19.8%)	53 (52.5%)	
Smoking^b^	6 (5.9%)	3 (3.0%)	.249
Alcohol^b^	3 (2.9%)	2 (2.0%)	.500
Physical activity^b^			
Low	72 (71.3%)	63 (62.4%)	.380
Moderate	26 (25.7%)	35 (34.7%)	
High	3 (3.0%)	3 (3.0%)	
Height^a^	163.58 ± 9.67	163.17 ± 7.50	.736
Body weight^a^	80.47 ± 17.23	73.73 ± 14.69	.003
Body mass index^a^	30.16 ± 6.45	27.75 ± 5.56	.005
Waist circumference^b^	93.58 ± 14.64	89.62 ± 13.74	.049
MIND diet score^a^	5.168 ± 1.525	8.797 ± 1.740	<.0001

Abbreviations: MIND, Mediterranean‐DASH (Dietary Approaches to Stop Hypertension) Intervention for Neurodegenerative Delay; RA, Rheumatoid Arthritis.

^a^ Data are presented as mean ± SD.

^b^ Data are presented as frequency (percent).

### 3.2 Association between MIND diet score and odds of RA

The crude and multivariate‐adjusted ORs and 95% CI for RA by level of following the MIND diet are presented in Table 2. After adjusting for various main covariates in the binary logistic regression test, participants with a higher MIND diet score had lower odds of RA in crude (OR: 0.194; 95% CI: 0.122, 0.311, *p* < .001), model I (OR: 0.067; 95% CI: 0.016, 0.283, *p* < .001), and model II (OR: 0.061; 95% CI: 0.013, 0.286, *p* < .001) analyses.

**TABLE 2:** Relation between adherence to MIND diet and odds of RA disease.

RA	OR (95% CI)	*p*‐value
Crude	0.194 (0.122, 0.311)	<.001
Multivariable‐adjusted model I	0.067 (0.016, 0.283)	<.001
Multivariable‐adjusted model II	0.061 (0.013, 0.286)	<.001

*Note*: Crude and multivariable‐adjusted OR and 95% CI for RA by MIND diet score were calculated using the Binary regression test.

Model I was adjusted for age, gender, education level, smoking, physical activity, and drugs.

Model II was adjusted for covariates in model I and BMI.

Abbreviations: CI, Confidence Interval; MIND, Mediterranean‐DASH (Dietary Approaches to Stop Hypertension) Intervention for Neurodegenerative Delay; OR, Odds Ratio; RA, Rheumatoid Arthritis.

### 3.3 Association between MIND diet score and stress oxidative biomarkers

As presented in Table 3, no remarkable correlation was found between the MIND diet and any of the oxidative stress factors (including GPx, TAC, MDA, and SOD; *p* > .05).

**TABLE 3:** Relationship between MIND diet score and oxidative stress factors in patients with RA.

Variables	Univariate model	*p*‐value	Multivariate model I	*p*‐value	Multivariate model II	*p*‐value
	β (95% CI)		β (95% CI)		β (95% CI)	
MDA (nmol/mL)	−0.034 (−0.076, 0.008)	.115	−0.029 (−0.076, 0.017)	.214	−0.028(−0.075, 0.019)	.239
SOD (mU/mL)	−0.037 (−3.907, 2.731)	.726	−0.091 (−5.113, 2.338)	.461	−0.087 (−5.132, 2.412)	.475
TAC (mmolFe^+2^/L)	−0.149 (−0.057, 0.010)	.162	−0.226 (−0.074, 0.001)	.055	−0.220 (−0.073, 0.003)	.067
GPx (mU/mL)	−0.054 (−3.969, 2.351)	.612	−0.059 (−4.413, 2.413)	.562	−0.073 (−4.623, 2.247)	.531

*Note*: Crude and multivariable‐adjusted OR and 95% CI for RA by MIND diet score were calculated using the linear regression test.

Model I was adjusted for age, gender, education level, smoking, physical activity, and drugs.

Model II was adjusted for covariates in Model I and BMI.

Abbreviations: CI, Confidence Interval; CRP, C‐Reactive Protein; GPx, Glutathione Peroxidase; MDA, Malondialdehyde; MIND, Mediterranean‐DASH (Dietary Approaches to Stop Hypertension) Intervention for Neurodegenerative Delay; OR, Odds Ratio; RA, Rheumatoid Arthritis; SOD, Superoxide Dismutase; TAC, Total Antioxidant Capacity.

### 3.4 Association between MIND diet score and disease activity

**FIGURE 1:**
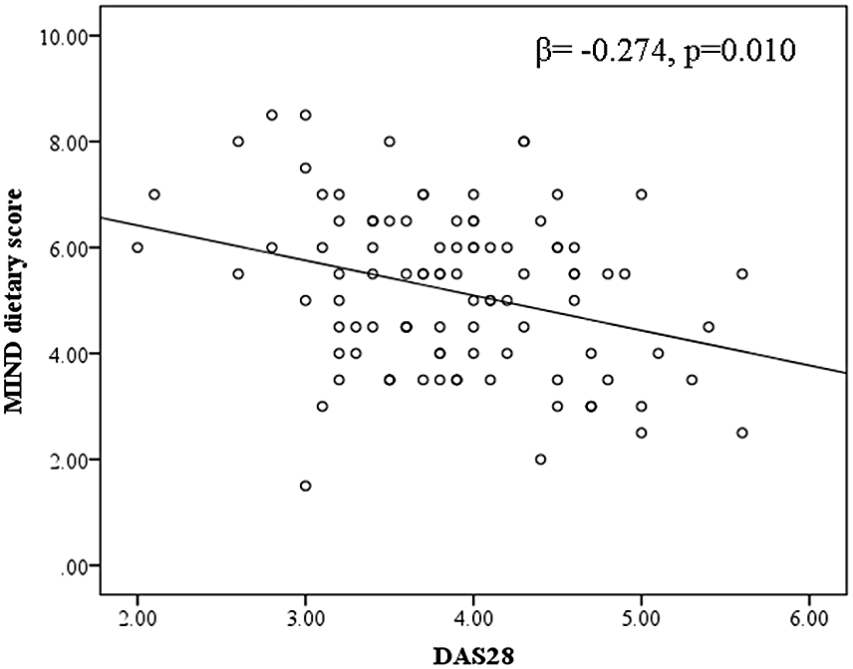
Association between the MIND dietary pattern and DAS‐28 index in patients with RA. In the current study, the DAS‐28 index was negatively and significantly correlated with the MIND diet score in patients with RA. DAS‐28, Disease Activity Score of 28 Joints; MIND, Mediterranean‐DASH Intervention for Neurodegenerative Delay; RA, Rheumatoid Arthritis.

As shown in Figure 1, the MIND diet score had a considerable inverse association with DAS28 analyses (β: −0.274; 95% CI: −0.220, −0.031, *p* = .010). Based on these findings, for each point increase in the MIND diet, the disease severity decreased by 0.247 points.

### 3.5 Association between MIND diet score and lipid profile

As presented in Table 4, after considering all the possible confounders (model II), a significant inverse association was observed between the MIND diet score and TG (β: −0.627; 95% CI: −0.087, −0.053, *p* < .001), TC (β: −0.368; 95% CI: −0.033, −0.010, *p* < .001), and LDL (β: −0.252; 95% CI: −0.035, −0.004, *p* = .015) levels. Also, a significant direct association was seen between HDL (β: 1.899; 95% CI: 0.382, 3.416, *p* = .015) and the MIND diet. Lower levels of FBG (β: −1.638; 95% CI: −2.650, −0.626, *p* = .002) and HA1C (β: −0.375; 95% CI: −0.184, −0.061, *p* < .001) had a significant association with higher adherence to the MIND diet. The MIND diet had no significant correlation with insulin and HOMA‐IR (*p* > .05).

**TABLE 4:** Relation between MIND diet score and metabolic factors in patients with RA.

Variables	Univariate model	*p*‐value	Multivariate model I	*p*‐value	Multivariate model II	*p*‐value
	β (95% CI)		β (95% CI)		β (95% CI)	
TG (mg/dL)	−0.681 (−0.093, −0.060)	<.001	−0.638 (−0.088, −0.054)	<.001	−0.627 (−0.087, −0.053)	<.001
LDL (mg/dL)	−0.235 (−0.033, −0.003)	.017	−0.249 (−0.034, −0.004)	.015	−0.252 (−0.035, −0.004)	.015
HDL (mg/dL)	1.935 (0.538, 3.333)	.007	1.904 (0.399, 3.409)	.014	1.899 (0.382, 3.416)	.015
TC (mg/dL)	−0.411 (−0.035, −0.014)	<.001	−0.371 (−0.033, −0.010)	<.001	−0.368 (−0.033, −0.010)	<.001
FBG (mg/dL)	−2.109 (−3.127, −1.092)	<.001	−1.642 (−2.646, −0.638)	.002	−1.638 (−2.650, −0.626)	.002
HbA1C	−0.440 (−0.203, −0.086)	<.001	−0.384 (−0.187, −0.064)	<.001	−0.375 (−0.184, −0.061)	<.001
Insulin (μIU/mL)	0.110 (−0.480, 1.528)	.302	0.056 (−0.850, 1.359)	.648	0.050 (−0.890, 1.345)	.686
HOMA‐IR	−0.003 (−0.047, 0.040)	.882	−0.012 (−0.061, 0.036)	.623	−0.014 (−0.063, 0.034)	.561

*Note*: Crude and multivariable‐adjusted OR and 95% CI for RA by MIND diet score were calculated using the linear regression test.

Model I was adjusted for age, gender, education level, physical activity, smoking, and drugs.

Model II was adjusted for covariates in Model I and BMI.

Abbreviations: CI, Confidence Interval; FBG, Fasting Blood Glucose; HbA1C, Hemoglobin A1C; HDL, High‐Density Lipoprotein cholesterol; HOMA‐IR, Homeostatic Model Assessment for Insulin Resistance; LDL, Low‐Density Lipoprotein cholesterol; MIND, Mediterranean‐DASH (Dietary Approaches to Stop Hypertension) Intervention for Neurodegenerative Delay; OR, Odds Ratio; RA, Rheumatoid Arthritis; TC, Total Cholesterol; TG, Triglyceride.

## 4 DISCUSSION

This is the first research to evaluate the correlation between following a MIND diet and oxidative stress biomarkers, metabolic factors, disease severity, and odds of disease in RA patients. Many potential benefits have been attributed to the MIND diet in disease prevention and health maintenance, mainly due to its plant‐based origins (Barnes et al., 2023). The findings of the present work demonstrated that patients with RA had lower adherence to the MIND diet than healthy individuals. Using each unit of the MIND diet reduced 94% of the odds of RA and 0.247 points the severity of the disease. Following the MIND diet was linked to lipid profile and some glycemic indices but did not correlate with oxidative stress factors.

Our results showed that the odds of RA were significantly lower in the higher scores of the MIND diet. Considering that this research is the first investigation that evaluates the correlation between the MIND diet and RA, the scientific discussion about the results is challenging. A series of recent studies showed that following a healthy food pattern with almost the same features as the MIND diet reduces RA risk (Alwarith et al., 2019; Gioia et al., 2020; Philippou & Nikiphorou, 2018). Our result is consistent with a cohort study that showed the Mediterranean diet may lower the risk of RA (Nguyen et al., 2021). Recent case–control research on Swedish adults, including 1721 individuals with RA and 3667 healthy people, observed a reverse correlation between the Mediterranean diet score and RA (Johansson et al., 2018). A prospective cohort study involving 174,638 female nurses with 913 incident RA cases did not indicate any correlation between the Mediterranean diet score and the chance of RA (20). In the mentioned research, the results cannot be generalized due to the consideration of only women and the population with high knowledge about health (Hu et al., 2015). Cross‐sectional research showed that following the DASH diet was less common in the RA cases than in the control group (Ghaseminasabparizi et al., 2021). Studies in other types of arthritis indicated a remarkable inverse correlation between following the DASH pattern and the incidence of osteoarthritis (Zhang et al., 2020). It seems that herbal‐based dietary patterns, which have high antioxidants and improve the intestinal microbiota (Picchianti Diamanti et al., 2020), may reduce the risk of RA. Also, since obesity can increase the chance of RA, plant‐based dietary patterns may reduce the chance of RA by decreasing obesity (Moroni et al., 2020).

The results of the present study showed that the MIND diet had a substantial inverse relationship with disease severity. In a study, Diamanti et al. reported that patients with a high Mediterranean diet score had a significantly lower disease severity than patients with a low or moderate Mediterranean diet score (Picchianti Diamanti et al., 2020). Also, Sköldstam et al. showed a remarkable reverse correlation between Mediterranean diet score and disease activity (Sköldstam et al., 2003). In addition, Abed et al. observed a significant reduction in DAS28 after 13 days of Mediterranean diet intervention in RA patients (Abendroth et al., 2010). The improvement of the patient's physical condition due to the use of high‐quality diets with high fiber and monounsaturated fat content and low saturated fat may be a reason for the reduced disease severity (Matsumoto et al., 2018).

Also, in this research, we considered the relationship between following the MIND diet and oxidative stress and found no remarkable relationship. According to evidence, plant‐based dietary patterns can improve oxidative stress in different diseases. For example, adherence to the DASH diet can reduce MDA levels and increase TAC levels in patients with diabetes and metabolic syndrome (Pirouzeh et al., 2020). Also, in a cross‐sectional study, Azzini et al. detected that higher following the Mediterranean diet was linked to lower levels of MDA in healthy populations (Azzini et al., 2011). However, consistent with our findings, some studies showed conflicting results. For example, Razavi Zade et al. (2016) reported that the 8‐week intervention with the DASH diet had no meaningful effect on the level of TAC in patients with non‐alcoholic fatty liver disease. Also, Ruggeri et al. did not observe a substantial correlation between the Mediterranean diet and SOD, GPX, and TAC in patients with Graves' disease (Ruggeri et al., 2021).

According to our findings, a higher MIND diet score was related to an improved lipid profile. The result is consistent with the Fateh et al. study, which showed that following the MIND diet reduced TG and increased HDL levels in obese people (Fateh et al., 2023). Also, Mohammadpour et al. (2020), in a cross‐sectional investigation, found a substantial positive correlation between following the MIND diet and HDL levels. Moreover, Khatibi et al. (2021) showed that following the MIND diet was associated with less hypercholesteremia. As mentioned earlier, the MIND diet emphasizes the consumption of berries (Hu et al., 2015). Berries are a rich source of flavonoids and anthocyanins. Flavonoids, as antioxidants, inhibit LDL oxidation (Calvano et al., 2019). Also, high fiber in the MIND diet may increase the attachment of cholesterol to bile acids, which lowers blood cholesterol. Also, clone bacteria ferment fibers and inhibit cholesterol synthesis by producing acetate and butyrates (Ojo et al., 2021).

We found that following a high MIND dietary pattern was associated with lower levels of FBG and HbA1C. Ardekani et al., in a cross‐sectional investigation, indicated that a higher MIND score was linked to greater insulin sensitivity but not to other metabolic risk factors (Ardekani et al., 2023). In this regard, a recent review study reported that high adherence to the Mediterranean diet may lower FBG levels (Magriplis & Chourdakis, 2021). A randomized controlled trial study in diabetic patients showed that adherence to the DASH pattern for 12 weeks caused a significant decrease in HbA1C (Daneshzad et al., 2022). The high fiber in these plant‐origin food patterns seems to help food digestion and reduce hunger by slowing down the stomach. Therefore, the rate of metabolism of carbohydrates to blood glucose decreases (Papamichou et al., 2019).

The main limitation of this study is its cross‐sectional design, which has less ability to demonstrate association than other observational studies like cohort studies. Also, FFQ has been used to evaluate food intake. Despite its high reliability, it depends on memory and may affect the accuracy of dietary data.

## 5 CONCLUSIONS

The findings indicate that following the MIND diet might be related to reduced disease activity and the odds of RA. It also improves the lipid profile and blood glucose status. However, further examinations are needed to support our findings.

## AUTHOR CONTRIBUTIONS

**Mahdieh Safaei:** Data curation (equal); formal analysis (equal); investigation (equal); methodology (equal); software (equal); writing – original draft (equal); writing – review and editing (equal). **Sorayya Kheirouri:** Conceptualization (equal); funding acquisition (equal); project administration (equal); supervision (equal); validation (equal); writing – review and editing (equal). **Mohammad Alizadeh:** Methodology (equal); project administration (equal); supervision (equal). **Amir‐Hossein Pirovi:** Methodology (equal); supervision (equal).

## FUNDING INFORMATION

The Tabriz University of Medical Sciences, Tabriz, Iran, provided the economic support.

## CONFLICT OF INTEREST STATEMENT

The authors have declared that no conflict of interest exists.

## ETHICS STATEMENT

This research was accepted by the ethics board of Tabriz University of Medical Sciences (IR.TBZMED.REC.1400.1149). Written consent was received from all subjects.

## Data Availability

The data that confirm the results of this research are available from the corresponding author upon reasonable request.
